# P-1096. AI-Assisted Fluorescent Visualization of Hand Hygiene Accuracy in a Real-World Hospital Study

**DOI:** 10.1093/ofid/ofaf695.1291

**Published:** 2026-01-11

**Authors:** changhua Chen, Mei-Ling Yang, Chew-Teng Kor, Chiu-Hsiang Liao

**Affiliations:** Changhua Christian Hospital, Changhua, Changhua, Taiwan (Republic of China); Changhua Christian Hospital, Changhua, Changhua, Taiwan (Republic of China); Changhua Christian Hospital, Changhua, Changhua, Taiwan (Republic of China); Changhua Christian Hospital, Changhua, Changhua, Taiwan (Republic of China)

## Abstract

**Background:**

Hand hygiene is a critical strategy in reducing healthcare-associated infections. While training and protocols are widely implemented, the actual accuracy of hand hygiene technique often remains unverified. This real-world study used AI-assisted fluorescent visualization (Figure 1) to objectively assess the accuracy of hand hygiene performance among healthcare workers and hospital visitors.

**Figure:**
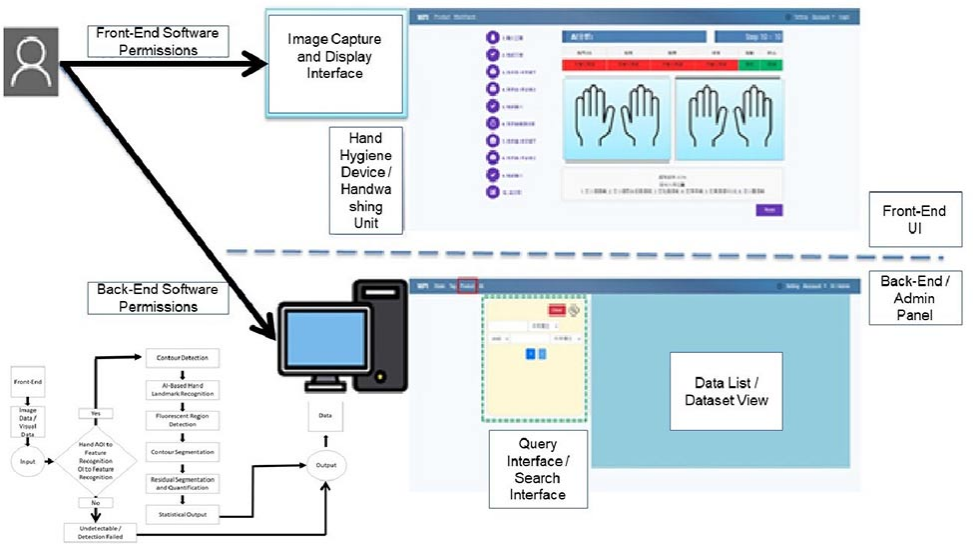
Integrated System Architecture and Workflow for AI-Assisted Assessment of Hand Hygiene Accuracy This schematic illustrates the comprehensive system framework employed to evaluate hand hygiene accuracy. The upper panel presents the front-end testing interface, where participants perform hand hygiene and fluorescent images are captured under controlled conditions. The lower-left segment outlines the procedural data flow, encompassing participant classification, regional hand mapping, and motion-specific segmentation. The back-end infrastructure integrates AI-powered image analysis, automated fluorescence quantification, and data visualization modules to support accuracy assessment across six critical anatomical hand zones.

**Figure:**
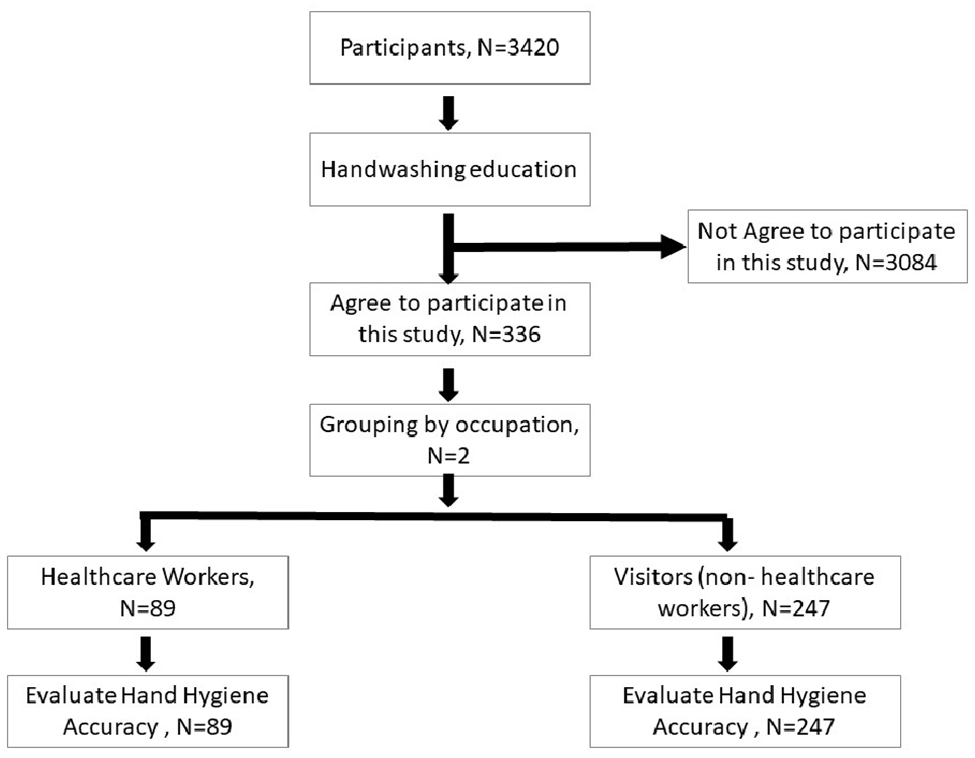
Participant Enrollment Flow and Occupational Group Stratification Enrollment flowchart summarizing participant recruitment and group assignment. Of 3,420 individuals approached, 336 consented to participate. Participants were stratified into two cohorts based on occupational status: healthcare workers (n=89) and non-clinical visitors (n=247). Both groups received standardized training based on the WHO six-step hand hygiene technique prior to accuracy evaluation using AI-assisted fluorescence analysis.

**Methods:**

The study was conducted in a hospital environment. Participants included healthcare professionals and non-medical individuals simulating visitors (Figure 2). All were trained in the WHO six-step hand hygiene method. Fluorescent lotion was applied to mimic microbial contamination. After hand hygiene, hands were examined under 365 nm UV light. AI-enhanced image analysis was used to evaluate six anatomical zones (fingertips, backs of fingers, interdigital spaces, backs of hands, finger pads, palms) for residual fluorescence. Accuracy was defined as complete removal of simulated contaminants in all zones. A one-sample binomial test was used to determine if ≥90% accuracy was achieved.

**Figure:**
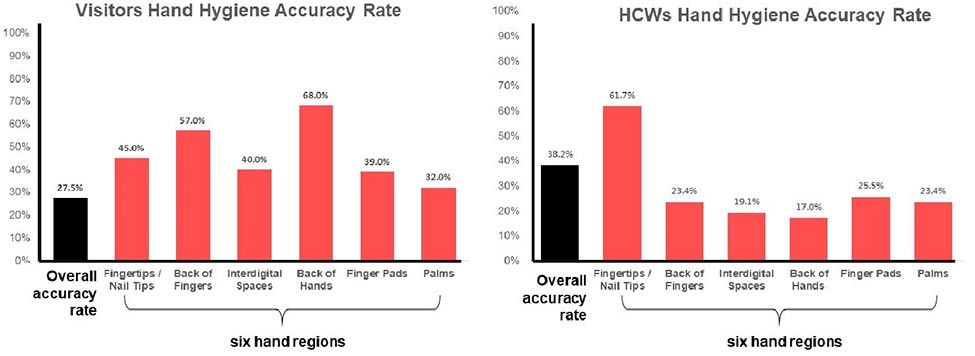
Comparative Hand Hygiene Accuracy Across Anatomical Regions in Healthcare Workers and Visitors Side-by-side bar charts illustrating hand hygiene accuracy by anatomical region. Among visitors (left), the highest accuracy was observed at the palms (57.0%), while the lowest occurred at the fingertips/nail tips (27.5%) and backs of fingers (40.0%). For healthcare workers (right), accuracy peaked at the backs of hands (61.7%), with notable deficiencies at the interdigital spaces (19.1%) and fingertips/nail tips (38.2%). Neither group achieved the predefined ≥90% accuracy threshold in any evaluated zone.

**Figure:**
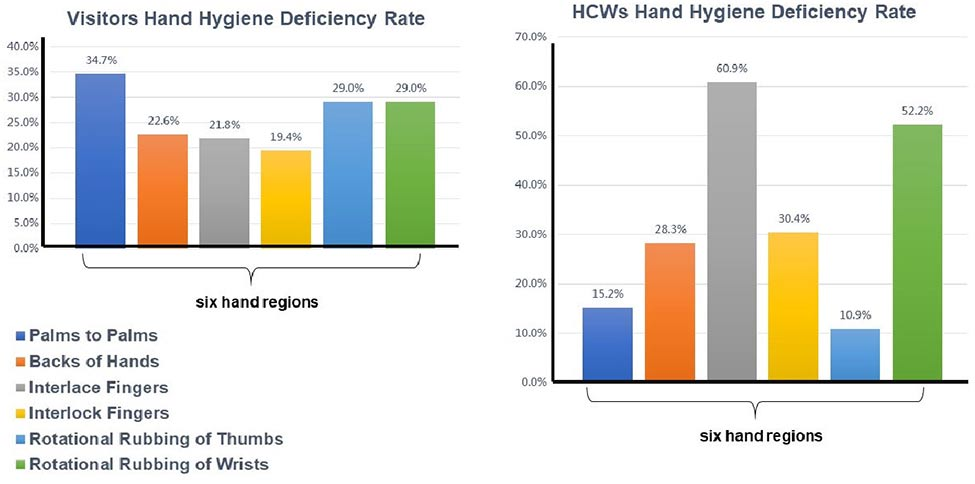
Anatomical Distribution of Hand Hygiene Deficiencies by Participant Group Bar graphs comparing the frequency of residual contamination across six hand regions in healthcare workers(left) and visitors(right). Interdigital spaces and fingertips were the most frequently missed zones in both groups. These consistent anatomical gaps underscore the limitations of current educational strategies and reinforce the need for precision-focused, feedback-enhanced hand hygiene interventions.

**Results:**

Neither group achieved the 90% accuracy threshold (p < 0.001). Healthcare professionals showed 38.2% overall accuracy, while visitors reached 45.0% (Figure 3). High-residue areas included fingertips, interdigital spaces, and backs of fingers (Figure 4). Motions most frequently associated with residuals were interlacing fingers, rotational wrist rubbing, and back-of-hand movements. AI analysis enabled precise identification of technique deficiencies linked to specific motions.

**Conclusion:**

This study revealed that, even with standardized instruction, hand hygiene technique accuracy remains low in both healthcare workers and visitors. AI-assisted fluorescent visualization provided an effective, objective method for identifying critical gaps in technique. These insights support the implementation of targeted, data-driven feedback interventions to improve the precision of hand hygiene practices in healthcare settings.

**Disclosures:**

All Authors: No reported disclosures

